# Supplementary material for: GATA4-targeted compounds induce apoptosis and diminish viability of hepatoblastoma cells
Source: PLoS One. 2026 Feb 11;21(2):e0342565. doi: 10.1371/journal.pone.0342565 (PMC12893608; doi:10.1371/journal.pone.0342565)
Supplement: S1 File — (PDF) [file pone.0342565.s001.pdf]

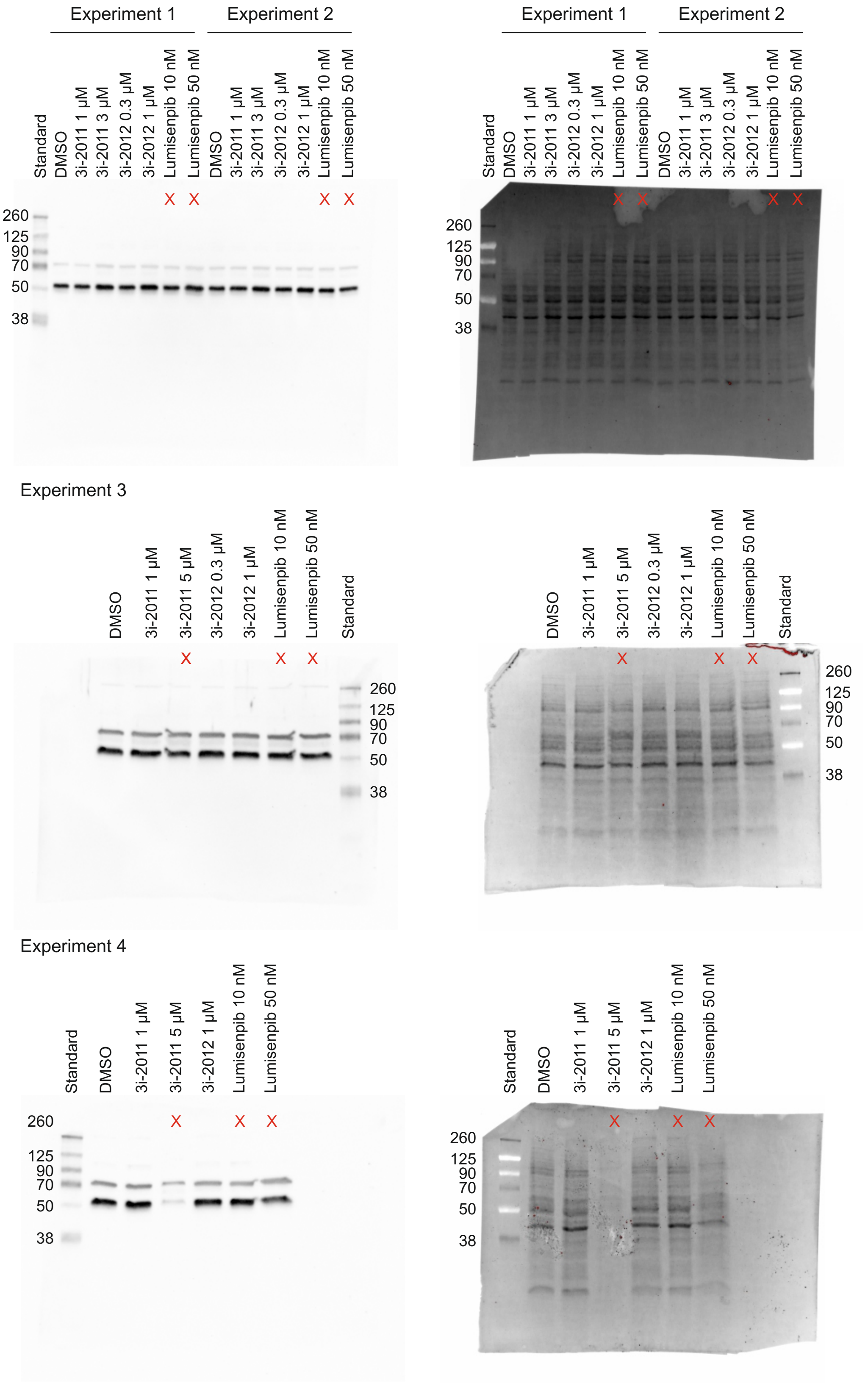

Raw blots of GATA4 detection blots (left panel) and total proteins blots (right panel). Supplementary Figure S4b was generated from the ”Experiment 1" of this figure. Samples marked with a red ”X” are not included in the final analyses. Protein bands were detected utilizing Enhanced Chemiluminescence detection kit and analyzed with Image Lab Software 6.0. Band intensity was normalized to total protein amount in each lane using stain-free technology.
