# Supplementary material for: GATA4-targeted compounds induce apoptosis and diminish viability of hepatoblastoma cells
Source: PLoS One. 2026 Feb 11;21(2):e0342565. doi: 10.1371/journal.pone.0342565 (PMC12893608; doi:10.1371/journal.pone.0342565)
Supplement: S1 Fig — Based on the structural analysis, we introduced a variable morpholine-extensions to the 3i-2000 compound scaffold, leading to the discovery of novel GATA4-modulators 4-(tert-butyl)-N-(3-(4-(2-morpholinoethoxy)phenyl)-1H-pyrazol-5-yl)benzamide (3i-2011), 4-(tert-butyl)-N-(3-(4-(3-morpholinopropoxy)phenyl)-1H-pyrazol-5-yl)benzamide (3i-2012) and 4-(tert-butyl)-N-(3-(3-methyl-4-(2-morpholinoethoxy)phenyl)-1H-pyrazol-5-yl)benzamide (3i-2013) showing an improved inhibitory activity in luciferase assays in comparison to original lead compound 3i-1000. (PDF) [file pone.0342565.s002.pdf]

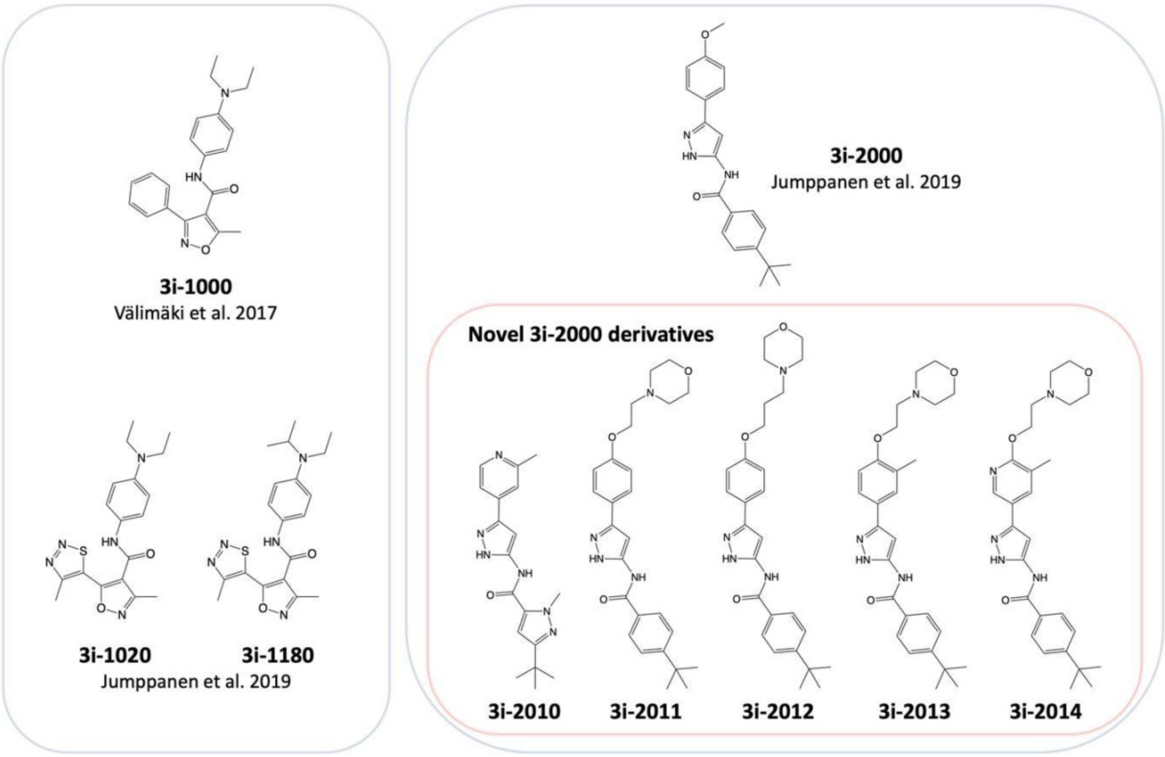

Supplementary Figure S1. Chemical structures of GATA4-acting compound 3i-1000 [1] and 3i-2000 [2] and their previously reported (3i-1020 [2], 3i-1180 [2]) or novel compound analogues (3i-2010-2014) investigated in this study. Based on the structural analysis, we introduced a variable morpholine-extensions to the 3i-2000 compound scaffold, leading to the discovery of novel GATA4-modulators 4-(tert-butyl)-N-(3-(4-(2-morpholinoethoxy)phenyl)-1H-pyrazol-5-yl)benzamide (3i-2011), 4-(tert-butyl)-N-(3-(4-(3-morpholinopropoxy)phenyl)-1H-pyrazol-5-yl)benzamide (3i-2012) and 4-(tertbutyl)-N-(3-(3-methyl-4-(2-morpholinoethoxy)phenyl)-1H-pyrazol-5-yl)benzamide (3i-2013) showing an improved inhibitory activity in luciferase assays in comparison to original lead compound 3i-1000.
