# Supplementary material for: GATA4-targeted compounds induce apoptosis and diminish viability of hepatoblastoma cells
Source: PLoS One. 2026 Feb 11;21(2):e0342565. doi: 10.1371/journal.pone.0342565 (PMC12893608; doi:10.1371/journal.pone.0342565)
Supplement: S2 Fig — Kinases found to bind are marked with red circles, where larger circles indicate higher-affinity binding. (PDF) [file pone.0342565.s003.pdf]

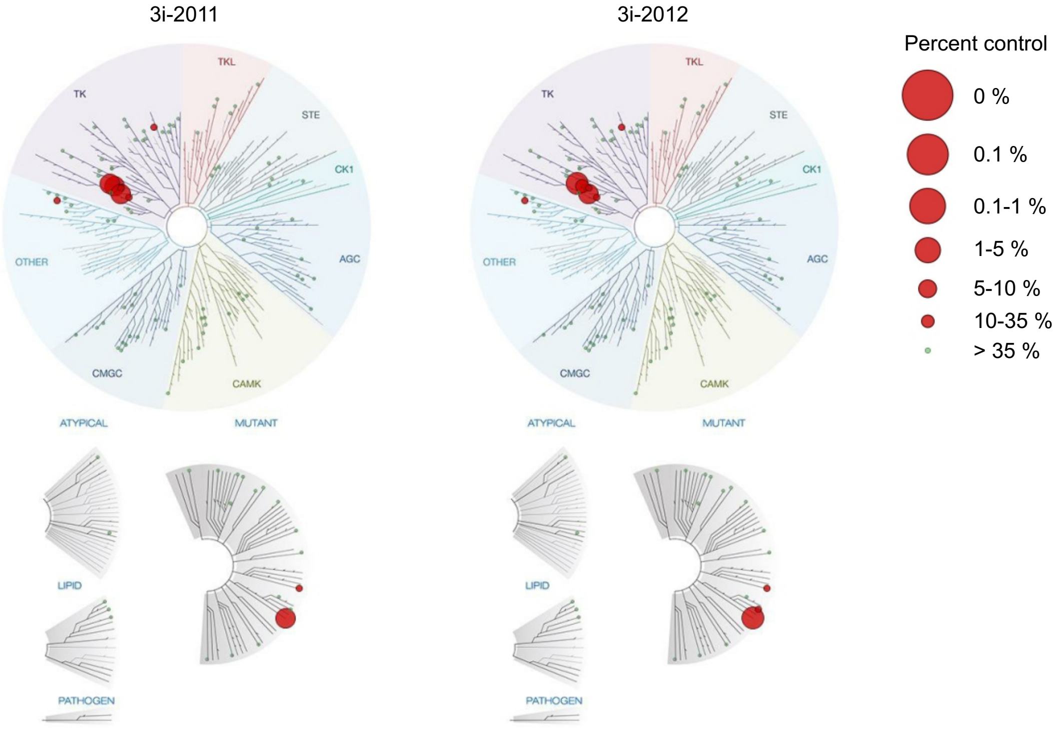

Supplementary Figure S2. TREEspot™ Interaction Maps of KINOMEscan™. Kinases found to bind are marked with red circles, where larger circles indicate higher-affinity binding.
