# Supplementary material for: GATA4-targeted compounds induce apoptosis and diminish viability of hepatoblastoma cells
Source: PLoS One. 2026 Feb 11;21(2):e0342565. doi: 10.1371/journal.pone.0342565 (PMC12893608; doi:10.1371/journal.pone.0342565)
Supplement: S3 Fig — Relative ATP concentration in HUH6 and HB-282 cells after 24 and 48 h treatment with 3i-2000 (a), 3i-2022 (b), and 3i-1180 (c). Results are presented as relative values of mean ± SD (N = 3). Dashed line indicates 50% cell viability. (PDF) [file pone.0342565.s004.pdf]

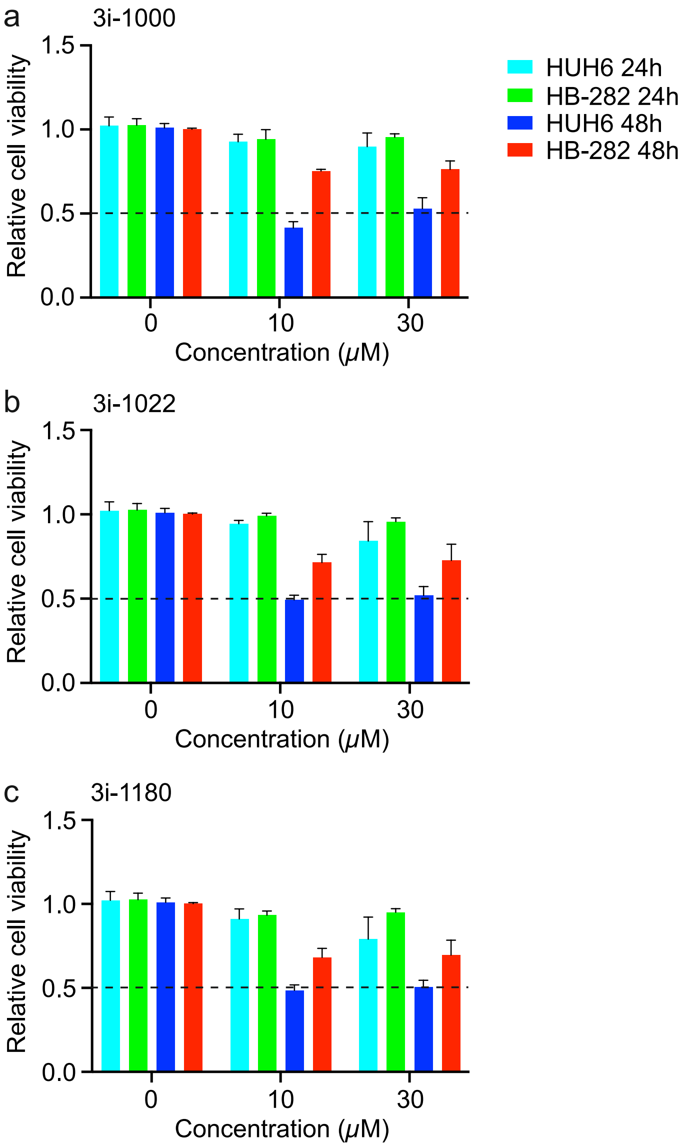

Supplementary Figure S3. The effect of small molecules on HB cell viability. Relative ATP concentration in HUH6 and HB-282 cells after 24 and 48 h treatment with 3i-2000 (a), 3i-2022 (b), and 3i-1180 (c). Results are presented as relative values of mean Å} SD (N = 3). Dashed line indicates 50% cell viability.
