# Supplementary material for: GATA4-targeted compounds induce apoptosis and diminish viability of hepatoblastoma cells
Source: PLoS One. 2026 Feb 11;21(2):e0342565. doi: 10.1371/journal.pone.0342565 (PMC12893608; doi:10.1371/journal.pone.0342565)
Supplement: S4 Fig — The Fig shows the average, + SEM and the result of each independent experiments n ≥ 3, except 3i-2011 3 μM n = 2 and for 3i-2012 0.3 μM in panel A n = 2. The original, uncropped raw images of the blots used to generate the western blot results are shown in S1 File. (PDF) [file pone.0342565.s005.pdf]

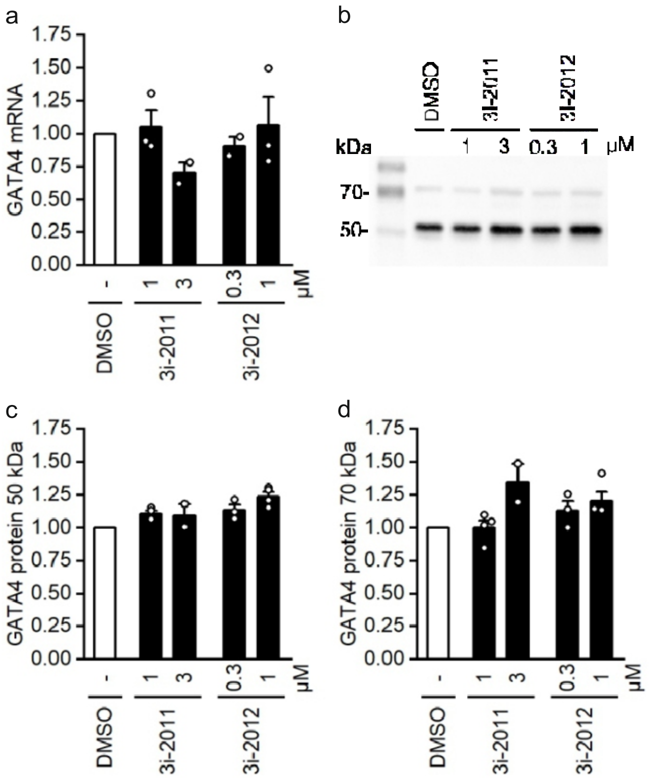


**Supplementary Figure S4.** Effect of small molecules on GATA4 mRNA (a) and protein levels (b-d) in HB-243 cells after 48 hours exposure. The figure shows the average, +SEM and the result of each independent experiments n≥3, except 3i-2011 3 µM n=2 and for 3i-2012 0.3 µM in panel A n=2. The original, uncropped raw images of the blots used to generate the western blot results are shown in Supplemental File S1_row_images.
