# Supplementary material for: GATA4-targeted compounds induce apoptosis and diminish viability of hepatoblastoma cells
Source: PLoS One. 2026 Feb 11;21(2):e0342565. doi: 10.1371/journal.pone.0342565 (PMC12893608; doi:10.1371/journal.pone.0342565)
Supplement: S5 Fig — The histograms show the number of nuclei and Hoechst staining intensity quantified from three technical replicates. (PDF) [file pone.0342565.s006.pdf]

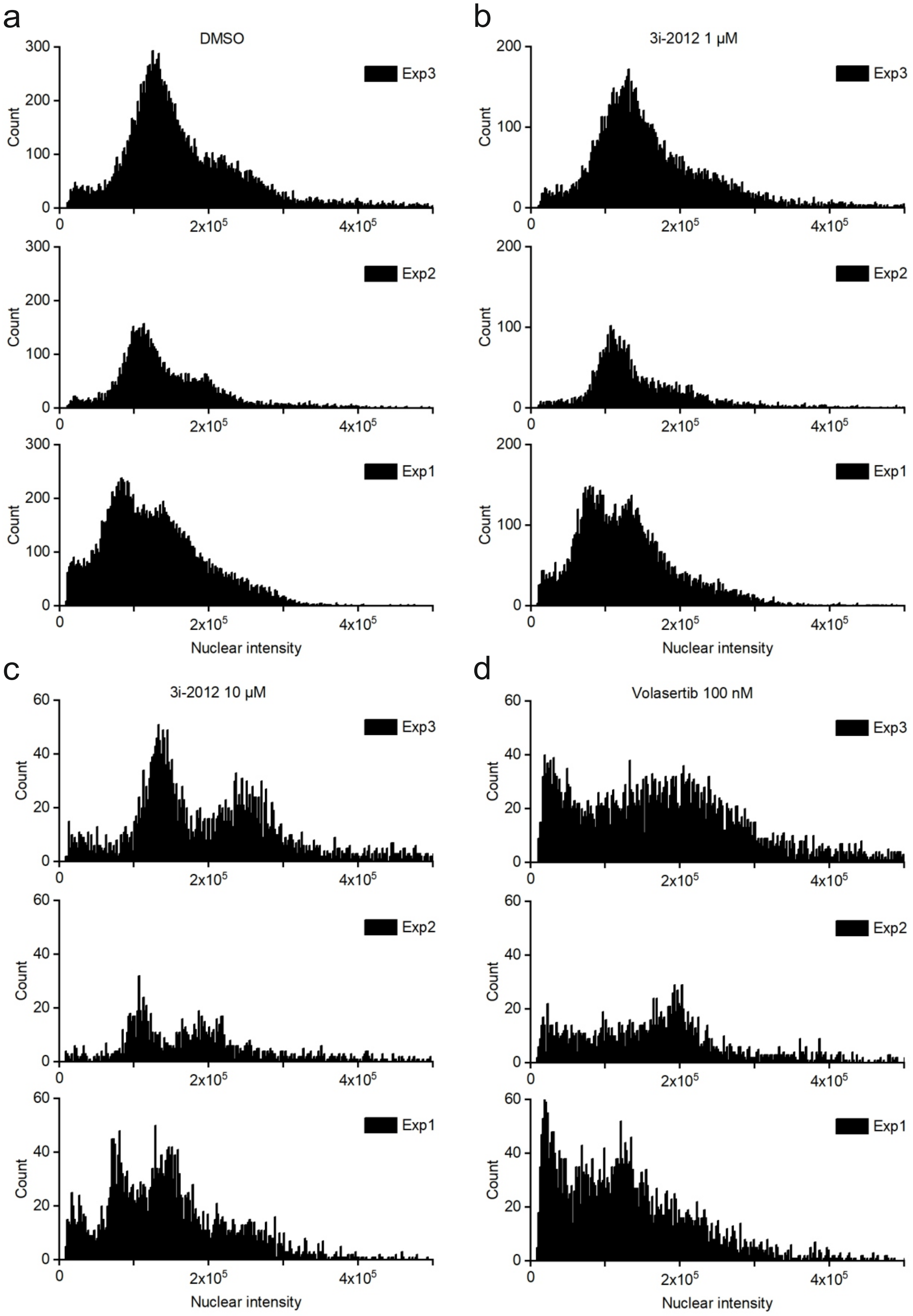

Supplementary Figure S5. High content analysis of the Hoechst staining intensity in nuclei after 24 hours exposure of DMSO (a), 1 µM 3i-2012 (b), 10 µM 3i-2012 (c), or volasertib (d) in HUH6 cells. The histograms show the number of nuclei and Hoechst staining intensity quantified from three technical replicates.
