# Supplementary material for: GATA4-targeted compounds induce apoptosis and diminish viability of hepatoblastoma cells
Source: PLoS One. 2026 Feb 11;21(2):e0342565. doi: 10.1371/journal.pone.0342565 (PMC12893608; doi:10.1371/journal.pone.0342565)
Supplement: S6 Fig — The box represents the interquartile range, and the whiskers represent the 1st and 4th quartile. The line inside the box is the median. (PDF) [file pone.0342565.s007.pdf]

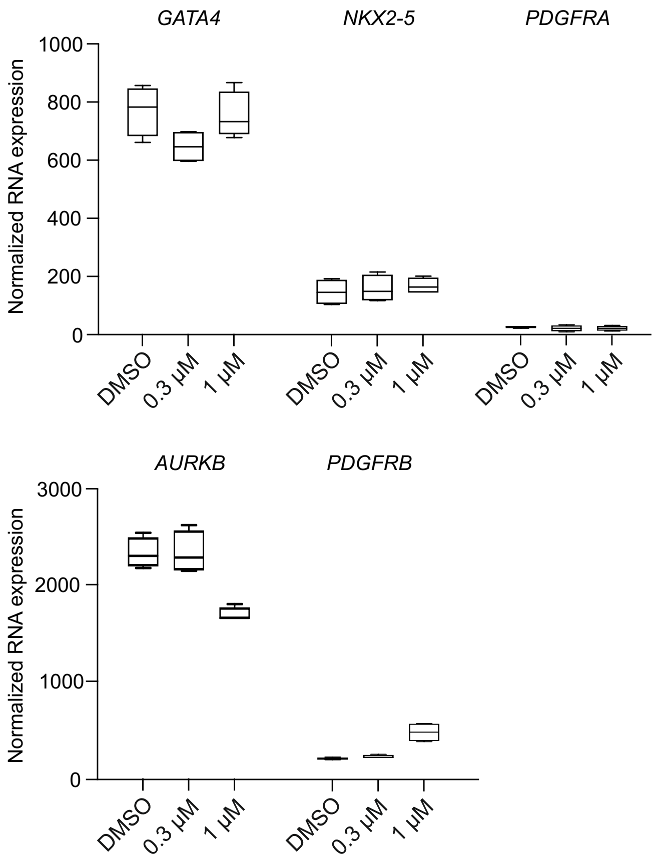

Supplementary Figure S6. The effect of 3i-2012 compound on *GATA4*, *NKX2-5*, and potential alternative targets identified by KINOMEscan assay on RNA expression in HB-243 cells. The box represents the interquartile range, and the whiskers represent the 1st and 4th quartile. The line inside the box is the median.
