# Supplementary material for: GATA4-targeted compounds induce apoptosis and diminish viability of hepatoblastoma cells
Source: PLoS One. 2026 Feb 11;21(2):e0342565. doi: 10.1371/journal.pone.0342565 (PMC12893608; doi:10.1371/journal.pone.0342565)
Supplement: S1 Table — Selected kinases were screened for target validation by Eurofins DiscoverX (Fremont, CA, USA). The results are presented as % from control. (PDF) [file pone.0342565.s008.pdf]

Supplementary Table S1. KINOMEscan™ results. Selected kinases were screened for target validation by Eurofins DiscoverX (Fremont, CA, USA). The results are presented as % from control.

**Target**	**3i-2010**		**3i-2011**		**3i-2012**		**3i-2013**		**3i-2014**	
**Gene symbol**	**1 µM**	**10 µM**	**1 µM**	**10 µM**	**1 µM**	**10 µM**	**1 µM**	**10 µM**	**1 µM**	**10 µM**
FLT3	43	3	0	0	0.15	0	3.2	0	19	0.65
FLT3(D835V)	79	12	70	17	78	21	91	41	89	68
FLT3(ITD)	59	20	0.1	0	20	0	27	8.7	35	20
FLT3(ITD.D835V)	86	47	87	67	98	87	94	90	100	100
FLT3(ITD.F691L)	100	69	94	60	87	63	91	82	89	78
FLT3-autoinhibited	91	52	33	4.1	56	14	82	54	80	62
KIT	81	16	7.7	0.2	12	0.15	61	14	16	0.25
PDGFRB	64	7.6	7.7	0.6	12	0.3	2.2	11	23	1.1

**Gene symbol**	**3i-2011 (10 µM)**	**3i-2012 (10 µM)**
ABL1(E255K) -phosphorylated	87	91
ABL1(T315I) -phosphorylated	94	100
ABL1-nonphosphorylated	75	71
ABL1-phosphorylated	88	88
ACVR1B	95	96
ADCK3	93	91
AKT1	100	100
AKT2	85	97
ALK	78	82
AURKA	87	77
AURKB	30	34
AXL	100	93
BMPR2	98	85
BRAF	91	100
BRAF(V600E)	95	100
BTK	98	93
CDK11	68	81
CDK2	100	97
CDK3	97	98
CDK7	83	78
CDK9	97	100
CHEK1	100	100
CSF1R	0.75	2.2
CSNK1D	98	92
CSNK1G2	100	99
DCAMKL1	78	74
DYRK1B	44	61
EGFR	74	69
EGFR(L858R)	100	100
EPHA2	99	100
ERBB2	73	85
ERBB4	69	76
ERK1	100	99
FAK	97	100
FGFR2	94	96
FGFR3	85	86
FLT3	13	11
GSK3B	100	100
IGF1R	100	97
IKK-alpha	90	83
IKK-beta	77	77
INSR	100	100
JAK2(JH1domain-catalytic)	69	78
JAK3(JH1domain-catalytic)	91	88
JNK1	84	78
JNK2	87	80
JNK3	85	73
KIT	0.2	0.1

**Gene symbol**	**3i-2011 (10 µM)**	**3i-2012 (10 µM)**
KIT(V559D,T670I)	56	30
LKB1	96	70
MAP3K4	98	93
MAPKAPK2	72	89
MARK3	70	73
MEK1	100	100
MEK2	100	100
MET	97	100
MKNK1	95	100
MKNK2	92	90
MLK1	91	75
p38-alpha	94	97
p38-beta	95	98
PAK1	100	100
PAK2	100	99
PAK4	100	100
PCTK1	100	99
PDGFRA	29	21
PDGFRB	0.6	0.35
PDPK1	100	100
PIK3C2B	92	90
PIK3CA	100	100
PIK3CG	100	100
PIM1	100	100
PIM2	100	100
PIM3	100	100
PKAC-alpha	100	100
PLK1	90	86
PLK3	79	70
PLK4	95	88
PRKCE	78	71
RAF1	100	100
RET	97	81
RIOK2	100	90
ROCK2	79	79
RSK2(Kin.Dom.1-N-terminal)	49	70
SNARK	78	71
SRC	100	100
SRPK3	87	84
TGFBR1	99	100
TIE2	66	82
TRKA	18	18
TSSK1B	85	92
TYK2(JH1domain-catalytic)	85	84
hULK2	100	93
VEGFR2	90	95
YANK3	78	90
ZAP70	100	97
