# Supplementary material for: GATA4-targeted compounds induce apoptosis and diminish viability of hepatoblastoma cells
Source: PLoS One. 2026 Feb 11;21(2):e0342565. doi: 10.1371/journal.pone.0342565 (PMC12893608; doi:10.1371/journal.pone.0342565)
Supplement: S2 Table — A panel of 32 bromodomain were screened for target validation by Eurofins DiscoverX (Fremont, CA, USA). The results are presented as % from control. (PDF) [file pone.0342565.s009.pdf]

Supplementary Table S2. BROMOscan™ results. A panel of 32 bromodomain were screened for target validation by Eurofins DiscoverX (Fremont, CA, USA). The results are presented as % from control.

**Gene symbol**	**3i-2012 (10 µM)**
ATAD2A	79
ATAD2B	94
BAZ2A	94
BAZ2B	89
BRD1	90
BRD2(1)	87
BRD2(2)	97
BRD3(1)	100
BRD3(2)	100
BRD4(1)	79
BRD4(2)	82
BRD7	91
BRD9	80
BRDT(1)	83
BRDT(2)	86
BRPF1	78
BRPF3	92
CECR2	78
CREBBP	100
EP300	94
FALZ	91
GCN5L2	88
PBRM1(2)	81
PBRM1(5)	97
PCAF	94
SMARCA2	100
SMARCA4	100
TAF1(2)	92
TAF1L(2)	88
TRIM24(PHD,Bromo.)	100
TRIM33(PHD,Bromo.)	78
WDR9(2)	89
